# Allegheny County Medical Society

**Published:** 1892-10

**Authors:** 


### *SOCIETY REPORT.*

### ALLEGHENY COUNTY MEDICAL SOCIETY.

*Scientific Meeting, June* 21, 1892.

REPORT OF A CASE OF STENOSIS AT THE AORTIC ORIFICE. HISTORY, DIAGNOSIS AND TREATMENT. BY DR. E. B. BORLAND.

This case is reported for your consideration and comment, on account of its being one of the rarer forms of organic change in the valve orifices of the heart, its murmur being frequently confounded with functional murmurs at the base, and its treatment being based on indications differing from the routine treatment of the other valve orifices or valve lesions.

Mrs. M., aged 63. Family history good, except some tendency to rheumatism in some of the other members of the family. She gives some history of chronic bronchitis in early life. Was married at the age of 46. Her first and only child was born after a difficult labor. General health good for the last 40 years preceding the present trouble, which began about two years ago, when she first noticed some shortness of breath on exertion and a little later on a short cough troubled her on rising in the morning. At intervals she complained of some uneasiness and pain in the region of the heart, and had frequent attacks of fainting. About one year ago she came under my observation with the following symptoms: Appetite poor, tongue moist, intensely red and deeply fissured. Tenderness over the stomach and some irregularity of the bowels. General appearance of anæmia and emaciation. Radial pulse so weak and compressible that it could only be counted with difficulty. Examination of the chest revealed the usual signs of chronic bronchitis. The impulse of the heart against the chest wall was not perceptible, and the position of the apex beat could not be determined by palpation on account of the feeble systole. The first sound of the heart, as heard over the mitral area, was very feeble, shorter than normal and absent in about one systole in twelve. The second sound, as listened to over the base, was preceded by a low pitched systolic murmur. The veins were more prominent than normal but no signs of dropsy. During last summer she suffered from the usual number of "fainting spells," followed by general prostration lasting from several hours to several days. These attacks were evidently excited by unusual physical exertion or chill. During their continuance the heart's action was weakened, the cough increased, cyanosis deepened and the tendency to syncope on rising much greater than usual. These attacks were treated with small doses of morphine or alcoholic stimulants. Digitalis was tried several times cautiously in small doses, but had to be discontinued on account of its increasing the dyspnœa and cyanosis, causing signs of pulmonary congestion and making the heart more irregular. Several preparations of iron were given to correct the general anæmia but were not well borne by the stomach. Teaspoonful doses of emulsion of cod liver oil given just before meals gradually improved her appetite and strength. The attacks of heart exhaustion became less frequent and less severe. In the early part of the winter she had an attack of acute bronchitis with a moderate rise of temperature, which yielded to the usual remedies. No other rise of temperature was noted, it being usually subnormal. Her condition at present is as follows: Tongue still red and fissured, digestion improved, urine normal in color and quantity, specific gravity 1022. No albumen or sugar, reaction decidedly acid, precipitate containing an excess of lime salts. The radial pulse is tardy, small and beats about sixty to the minute, but is noticeably stronger than it was a year ago. The percussion wave of its tracing has a low amplitude, the ascent is more gradual, the apex more rounded and the descent more gradual than normal. No dicrotic waves can be detected with the finger, and there is no visible pulsation in any of the larger arteries. The pulse intermits when the cardiac sounds are absent. The temperature in the afternoon is about 97.5 F.

The physical signs of bronchitis have almost disappeared, but the respirations are still about twenty per minute. The cough is absent except on rising in the morning.

The area of percussion dullness over the heart cannot positively be determined on account of the mammary gland. There is no impulse in the fifth intercostal space either on inspection or palpation. The apex probably strikes the sixth rib. The first sound, as listened to over the mitral area, is shorter, higher in pitch, and has lost its booming quality. Over the aortic area, during systole, a harsh murmur is heard which is transmitted to the subclavian and carotid arteries. No sound is audible over the aortic area at the time of the closing of the semilunar valves, but over the pulmonary valve area the closing of the semilunar valves of the right heart can be heard distinctly.

The clinical history of this case is one of atheroma of the aortic orifice, coronary arteries, and probably, to some extent, of all the larger arteries, with infiltration of lime salts, particularly around the aortic orifice. The age of the patient is in favor of atheromatous changes. After the aortic valves close there is no sign of a regurgitant murmur. This is evidence that the free edges of these valves approximate, and that there are no vegetations on their ventricular surfaces. The murmur heard over the aortic area during the passage only of the blood from the left ventricle to the aorta, is evidence of narrowing of the orifice or thickening of the bases of the valves so they cannot be pressed into the sinuses of Valsalva, or both. The imperfect nutrition of the heart is shown by its feeble systole, intermittent pulse and lack of complete compensatory hypertrophy. These are signs that the openings into the coronary arteries are either obstructed or these vessels are in a condition of sclerosis.

The irregular intermittent action of the heart is not a necessary factor of simple aortic stenosis, but is considered characteristic of diseased coronary arteries.

The aortic direct murmur is more likely to be mistaken for functional murmurs than any other abnormal sounds. The latter is most frequently heard over the base during systole, and the anæmia and emaciation in this case would seem to favor the functional murmur. In simple anæmia murmurs the frequency and force of the heart's action increases during mental excitement or physical exertion; or, in other words, there is a marked temporary palpitation. In this case the heart's action is weakened and not noticeably increased in frequency under similar conditions. The pulse of anæmia is not only more frequent but is large and soft. In its tracing the percussion wave has a higher amplitude; the ascent is steeper; the apex pointed and the descent rapid. While the general health of this patient has improved and the other valves can be heard closing more distinctly than a year ago, the murmur has increased in intensity and pitch instead of diminishing. Functional murmurs are heard over the pulmonary as well as over the aortic areas but do not replace the normal sounds.

The use of digitalis was not permissible in this case on account of its main action being the stimulation of the cardiac inhibitory center, nerve, nerve endings and ganglia; thus slowing the heart still more by increasing the length of the diastole which robs the brain of sufficient blood during the intervals between the systoles to maintain consciousness and vital action. Further, digitalis markedly stimulates the vaso-motor nerve apparatus and contracts particularly the arterioles and delivery vessels, thus increasing the obstruction in front of the weakened, badly nourished left ventricle.

The treatment of the periods of heart exhaustion was based on two indications: Stimulation of the cardiac motor and accelerator nerves and ganglia, and removing as far as possible the obstruction to the outflow of the blood into the peripheral circulation. Morphine in small repeated doses (gr. 1-16 to 1-8) evidently increases the force and frequency of the heart by stimulating the cardiac motor and accelerator nerve apparatus. It relieves obstruction in front of the heart by relaxing the voluntary muscular system and dilating the vessels of the skin. Its action in congesting the brain relieves the tendency to syncope. It also depresses the respiratory center, and slows respiration which seemed beneficial in this case on account of the disproportion between the respiratory movements and the pulse beats. Alcohol, in small repeated doses, stimulates first reflexly and later directly the cardiac motor and accelerator nerve apparatus, which increases the frequency and power of the heart's action. It also depresses the vaso-motor ganglia and center, thus dilating the peripheral vessels, causing the blood to flow more rapidly through them, and thus supplying the brain with sufficient blood.

In the general treatment cod liver oil was given to increase the nutrition of the heart muscle.

DR. LANGE: The case reported by Dr. Borland is very interesting, inasmuch as it is reported as a case of pure aortic stenosis, which condition is exceedingly rare. Stenosis alone is an exceedingly rare form of the disease, so exceedingly rare that when met with the physician is at a considerable loss as to the advisability of the usual remedies for loss of compensation, which, however, from the report in Dr. Borland's case, did not exist. This patient, if I understand the description of the case correctly, was, and for many years has been, an anæmic. The patient never gave any indication of rheumatism, one of the most common causes of valvular disease, and it is no evidence, although she came from a rheumatic family, that she had it. The fact that this patient could not take digitalis is interesting, for when a patient has loss of compensation and cannot take digitalis, then the prognosis is extremely bad. Of course we have our little list, digitalis, strophanthus, caffeine and convallaria, but in spite of this list, which might be increased by remedies which are not on my tongue, when a patient cannot take digitalis, then the prognosis is extremely bad.

There are two classes of patients who cannot take it, and the case reported by Dr. Borland to one of these classes properly belongs. The patients who cannot take it are those who have aortic insufficiency with the large, jumping, bounding pulse of aortic insufficiency; the patient cannot usually take digitalis, but when we do give it such a patient requires particularly close attention. The other class of patients who cannot take it are those whom it nauseates, and if persisted in it would throw them into colic. In the case reported by Dr. Borland the symptoms did not indicate the administration of digitalis. There was no loss of compensation. It is stated she had some shortness of breath. That is probably always present in such patients, even where dropsy has disappeared. Shortness of breath is a common thing with patients before the appearance of dropsy.

DR. W. C. SHAW: In some cases of examination for life insurance I have noticed where the party has had no history of rheumatism at the time of examination, on questioning his parents at home he was found to have had rheumatism when an infant, and that often accounts for the fact of valvular lesions without a history of rheumatism. Rheumatism in infants occurs oftener, I believe, than it is recognized. I have in my mind now an agent of an insurance company who had valvular disease of the heart, and never supposed he had rheumatism until after some years, when he had been rejected once or twice, his mother told him he had had rheumatism when an infant.

DR. BORLAND: Dr. Lange must have misunderstood my report of the case in regard to compensation. There were signs of loss of compensation when I examined the case first, and at the present time there are slight signs; also a number of signs of dilatation are present at this time. The doctor also spoke of not using digitalis in aortic insufficiency, though it was indicated in aortic stenosis. My experience in aortic insufficiency has been that it will bear digitalis better than the obstruction of the aortic orifice. Rapid pulse with low tension is, as I understand it, an indication for the use of digitalis.

